# L’arthrite tuberculeuse isolée du genou, un diagnostic difficile chez l’adolescent: rapport de cas

**DOI:** 10.11604/pamj.2020.37.225.26470

**Published:** 2020-11-06

**Authors:** Rachid Benchanna, Amine Benjelloune, Zidane Abdelafatah, Adil Arsalane, Hicham Janah, Jamal Oujaber, Meriem Boui, Ikram Samri, Rachid Bouchentouf

**Affiliations:** 1 Service de Pneumologie de l´Hôpital Militaire Avicenne Marrakech, Marrakech, Maroc,; 2 Service de Chirurgie Thoracique de l´Hôpital Militaire Avicenne Marrakech, Marrakech, Maroc,; 3 Service de Radiologie de l´Hôpital Militaire Avicenne Marrakech, Marrakech, Maroc

**Keywords:** Arthrite, tuberculose, Xpert Mtb Rif, prélèvement, rapport de cas, Arthritis, tuberculosis, Xpert MTB/RIF, sample collection, case report

## Abstract

La tuberculose est un problème majeur de santé publique au niveau mondiale. La forme ostéo-articulaire est rarissime, elle est dominée par la localisation vertébrale dans la moitié des cas. En raison de la symptomatologie qui est longtemps insidieuse d´une part, et de la difficulté d´isolement de l´agent pathogène d´autre part, le diagnostic de l´arthrite tuberculeuse isolée est souvent difficile et tardif. Nous rapportons une nouvelle observation d´arthrite tuberculeuse du genou chez une adolescente, dont le délai entre les premières manifestations cliniques et le diagnostic de certitude par mise en évidence du génome du Mycobacterium tuberculosis par l´Xpert MTB/RIF était de huit mois. A travers ce cas, nous insistons sur l´intérêt du prélèvement bactériologique et des méthodes de détections de la biologie moléculaire dans le diagnostic précoce et certains de l´arthrite tuberculeuse.

## Abstract (en)

Tuberculosis is a major global public health problem. Osteoarticular tuberculosis is very rare. In half of the cases it affects the vertebrae. Symptoms are insidious over a prolonged period and isolation of the pathogen is difficult, mostly leading to difficult and delayed diagnosis. We here report a new case of tuberculous arthritis of the knee in a teenager in whom the mean time between symtom onset and definitive diagnosis based on the detection of Mycobacterium tuberculosis genome by Xpert MTB/RIF test was eight months. This study emphasizes the importance of bacteriological sampling and diagnostic methods by molecular assay in early and definitive diagnosis of tuberculous arthritis.

## Introduction

La tuberculose est un problème majeur de santé publique au niveau mondiale. Ces dernières années l'Organisation mondiale de la Santé (OMS) estime 10 millions de nouveaux cas annuel avec 1,5 millions de décès dont 251 000 porteurs du VIH et 480 000 cas de tuberculose multi résistante [1]. La tuberculose ostéo-articulaire est la quatrième localisation extra-pulmonaire. Son diagnostic ne pose pas de problème en cas de localisation pulmonaire bacilliforme concomitante, tandis qu´il est souvent difficile et tardif en cas d´atteinte articulaire isolée [2]. Il s´agit d´une forme pauci bacillaire dont le diagnostic a bénéficié récemment des progrès de la biologie moléculaire notamment l´Xpert MTB. Nous rapportons une nouvelle observation d´une arthrite tuberculeuse isolée du genou chez une adolescente dont le diagnostic était tardif et qui a été posé par la mise en évidence du génome du complexe *tuberculosis*.

## Patient et observation

Il s´agit d´une patiente âgée de 19 ans sans antécédent pathologique particulier consultant pour une arthrite chronique du genou gauche. L´histoire de la maladie remonte à 08 mois par l´installation brutale d´une arthrite fébrile du genou gauche, avec gonalgie, tuméfaction, rougeur avec limitation de l´extension de l´articulation et un signe de glaçon positif. La ponction du liquide synoviale a montré une cytologie à 80% de polynucléaires neutrophiles avec un examen direct et une culture stérile. La patiente était mise sous antibiotiques à base d´amoxicilline protégée avec une dose de 3g par jours pendant 15 jours, associé à 80 mg/24 heures de gentamicine pendant 07 jours avec une mise en décharge du genou gauche pendant une semaine et une mobilisation précoce. L´évolution était initialement favorable avec apyrexie, régression des signes inflammatoire avec persistance d´une gonalgie d´allure inflammatoire. Trois mois plus tard la patiente présentait une tuméfaction de la même articulation augmentant progressivement de volume avec fistule et issus purulent dans un contexte d´amaigrissement et d´anorexie.

**Figure 1:**
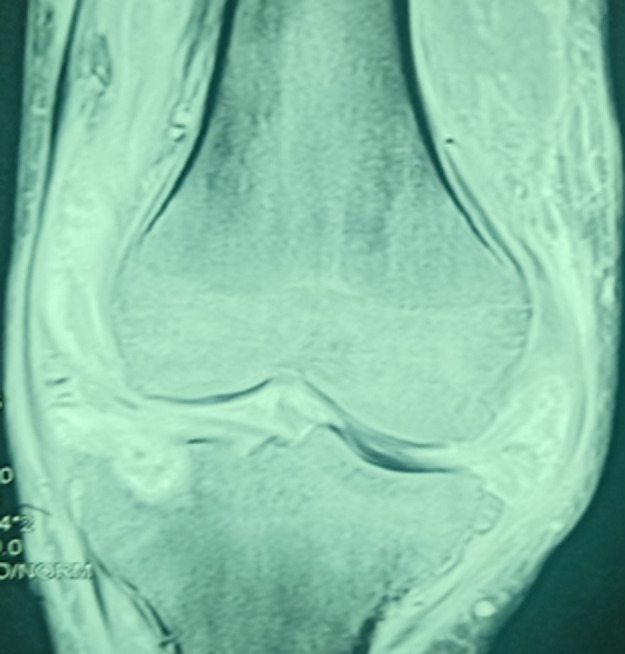
IRM du genou gauche en coupe coronale montrant la plage d´ostéolyse du plateau tibiale

**Figure 2:**
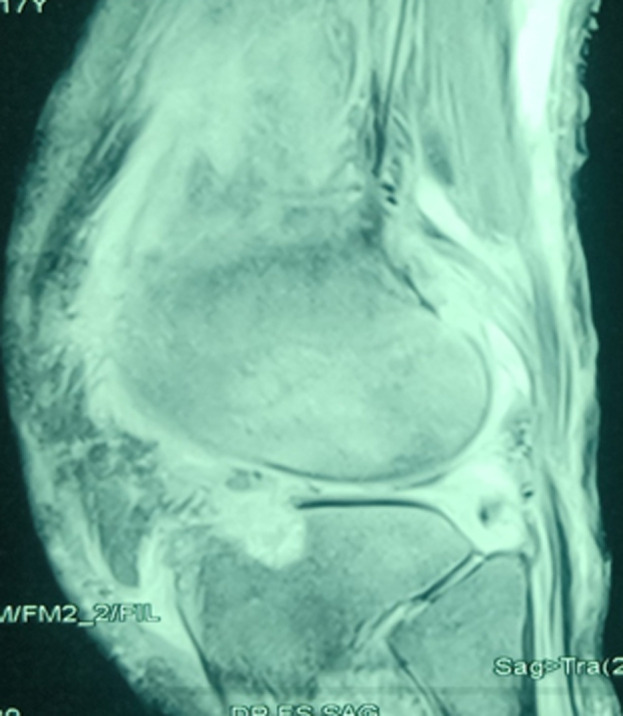
IRM en coupe sagittale du genou gauche montrant la profondeur de l´ostéolyse

Un bilan biologique a été réalisé et qui a montré un syndrome inflammatoire non spécifique avec une protéine C réactive (CRP) à 30 mg/l et une VS à 20 mm la première heure. La Tomodensitométrie (TDM) du genou nous a montré une ostéolyse en plage ostéochondrale du plateau tibiale externe associé à un épaississement synoviale diffus de l´articulation prenant le contraste (Figure 1, Figure 2). On a décidé devant ce tableau clinique trainant de refaire une ponction écho guidée qui a ramené un liquide synoviale jaune citrin séro-fibrineux dont la formule leucocytaire est à prédominance lymphocytaire. On a recherché le *Model-Based Testing* (MBT) par technique de l´Xpert MTB/RIF qui est revenu positif sensible à la rifampicine. On a complété notre bilan par une sérologie VIH qui est revenu négative, avec la recherche d´autres localisations tuberculeuses notamment pulmonaire. La radiographie du thorax était sans particularité, et la recherche du BK à l´expectoration par Xpert Mtb Rif était négative. La patiente était mise sous traitement médical sous le protocole 2RHZE/7RH (Rifampicine, Isoniazide, Pyrazinamide, Ethambutol) pendant neuf mois avec une rééducation et une mobilisation précoce de l´articulation. L´évolution était favorable.

## Discussion

La tuberculose est la principale cause de décès lié à une maladie infectieuse curable [3], et la première maladie opportuniste chez les patients vivants avec le VIH avec un taux de mortalité plus élevé que chez les tuberculeux séronégatifs [4]. Elle demeure un problème majeur de santé publique malgré la baisse du taux de mortalité de 47% entre 1990 et 2015 [5]. L´Afrique subsaharienne représente 25% de l´incidence mondiale avec près de 2 millions de tuberculeux [6]. La tuberculose ostéo-articulaire est une forme très ancienne, elle a été retrouvée chez les momies égyptiennes datant plus de 3400 ans avant Jésus-Christ [7]. La localisation ostéo-articulaire représente 10 à 20% des localisations extra-pulmonaires dont le siège vertébral est le plus fréquent [8]. La tuberculose est une maladie d´immunodépression cellulaire avec déplétion lymphocytaire, en l´occurrence la séropositivité au VIH ou la corticothérapie au long court.

L´atteinte osseuse résulte souvent d´une dissémination hématogène à partir d´un foyer de primo-infection, dont le rôle d´un traumatisme local a été suggéré [9]. En raison de la présentation clinique insidieuse en non spécifique, le délai diagnostique est souvent long avec un retentissement fonctionnel articulaire majeur. La radiographie peut montrer une atteinte destructive avec une ostéolyse, un pincement de l´interligne articulaire qui peut évoluer vers une déformation articulaire, une luxation ou l´apparition d´abcès froids à des stades évolués. La TDM peut être plus précise pour démonter le bilan lésionnel osseux notamment les séquestres. L’imagerie par résonance magnétique (IRM) peut retrouver précocement une synovite avec un bilan lésionnel précoce et extra-osseux [8]. Le diagnostic de la tuberculeuse reste bactériologique malgré la difficulté de l´isolement de l´agent pathogène dans les formes extra-pulmonaires qualifiées comme pauci-bacillaires en particulier l´atteinte ostéo-articulaire. En l´absence de ce diagnostic de certitude, la présence d´autres localisations notamment pulmonaire permet facilement de rattacher l´atteinte osseuse à l´origine tuberculeuse. En l´absence d´une forme pulmonaire bacilliforme associée, le diagnostic devient difficile.

L´apport des nouveaux moyens de diagnostiques de biologies moléculaires, ont révolutionnés le diagnostic bactériologique de la tuberculose. L´Xpert MTB/RIF est une technique de PCR en temps réel développé en 2011, dont les conditions de réalisation semblent moins complexes qu´aux autres techniques de biologie moléculaire, notamment le test d´hybridation inverse sur bandelette [10]. Il permet dans une seule cartouche un traitement de l´échantillon, la détection du gène robe et sa mutation (résistance à la rifampicine) ainsi que l´amplification enzymatique. Sa sensibilité (100 bacilles/ml) reste inférieure à celle de la culture (10 bacilles/ml) dont les conditions de réalisations et le délai d´obtention des résultats (03 à 08 semaines), laissent préférer cette méthode moléculaire qui est plus rapide et d´autre part plus sensible que l´examen direct classique [10].

## Conclusion

La tuberculose ostéo-articulaire isolée est de diagnostic difficile avec des signes cliniques et radiologiques peu spécifiques d´évolution subaiguë ou chronique à l´origine d´un retard diagnostique et des séquelles fonctionnelles non négligeables. A travers notre nouvelle observation, nous insistons sur l´intérêt du prélèvement à visée bactériologique devant toute arthrite d´évolution chronique, avec la recherche du *Mycobacterium tuberculosis* de préférence par les nouvelles méthodes de biologies moléculaires qui ont un délai de réponse réduit par rapport à la culture. Néanmoins, malgré l´absence de détection de l´agent pathogène, il est important de retenir le diagnostic devant des éléments de présomption notamment les tests IGRAS (interféron Gamma release assays), et démarrer un traitement précoce, seul garant d´une évolution favorable sans séquelles fonctionnelles redoutables.
